# Biochemical and Antioxidant Characteristics of *Chlorococcum oleofaciens* (Chlorophyceae, Chlorophyta) under Various Cultivation Conditions

**DOI:** 10.3390/plants13172413

**Published:** 2024-08-29

**Authors:** Irina Maltseva, Aleksandr Yakoviichuk, Svetlana Maltseva, Svetlana Cherkashina, Maxim Kulikovskiy, Yevhen Maltsev

**Affiliations:** 1 Faculty of Natural Sciences, Melitopol State University, 72312 Melitopol; maltseva-irina22@yandex.ru (I.M.); alex.yakov1991@gmail.com (A.Y.);; 2 K.A. Timiryazev Institute of Plant Physiology RAS, IPP RAS, 127276 Moscow, Russia; svetadm32@gmail.com (S.M.); max-kulikovsky@yandex.ru (M.K.)

**Keywords:** antioxidant activity, carotenoids, chlorophylls, enzymes, green algae, nitrogen and phosphorus deprivation, vitamins

## Abstract

**Abstract:** The functional state of enrichment cultures of the Chlorophycean strain *Chlorococcum oleofaciens* CAMU MZ–Ch4 under various cultivation conditions was studied. Experiments with different aeration conditions, cultivation durations, and nitrogen and phosphorus concentrations in the medium were carried out to evaluate the growth dynamics of the strain and its biochemical characteristics. The contents of chlorophylls, carotenoids, proteins, lipids, retinol, α-tocopherol, ascorbic acid, phenolic compounds, lipid peroxidation products, antioxidant enzymes (glutathione peroxidase, catalase, superoxide dismutase), and succinate dehydrogenase activity were measured. The lipid content on the fully supplemented Bold’s basal medium increased to 381.03 mg g^−1^ dry weight at the late stationary growth phase. This value is 1.3–2.8 times higher than in other experiments. The use of aeration was associated with an increased content of proteins at 283.56 mg g^−1^ and of carotenoids at 2.12 mg g^−1^. Also, cultures at the early stationary growth phase with aeration showed the ability to accumulate phenolic compounds and ascorbic acid in amounts up to 0.32 mg g^−1^ and 0.19 mg g^−1^. The 74-day-old cultures had the highest contents of retinol (0.16 mg g^−1^) and α-tocopherol (0.68 mg g^−1^). Growth in nitrogen- and phosphorus-depleted media increased catalase and superoxide dismutase activity. A comprehensive analysis of all data showed that the antioxidant defence system is stress-resistant and flexible under varying aeration conditions and nitrogen and phosphorus availabilities. Thus, the strain CAMU MZ–Ch4 can be considered a potential producer of lipids, pigments, proteins, and vitamins under various culturing conditions.

## 1. Introduction

Microalgae are a polyphyletic and diverse group of organisms that inhabit various aquatic and terrestrial biotopes. Recent studies show that many types of microalgae are sources of valuable compounds in demand for different economic activities [1,2,3]. Higher production costs compared to those for non-native feedstocks deter mass industrial microalgae biomass growth today [4]. To improve the profitability of production, many research efforts are focused on the search for new high-yield species or strains of microalgae in the natural ecosystem, as well as the development of strategies to enhance the synthesis of valuable compounds, including through the use of abiotic stresses [5,6,7].

Stress has been reported to affect the growth and productivity of microalgae adversely and hence inhibit the production of various compounds [8]. The problem of maintaining high biomass productivity and the synthesis of biotechnologically valuable compounds has not yet been solved. It is suggested that to manage the biosynthesis of beneficial compounds, it is essential to understand the interactions underlying the maintenance of cellular homeostasis by microalgae and the coordination of responses to external stresses.

During evolution, microalgae formed a network of metabolic reactions—an antioxidant system (AOS) aimed at reducing the damaging effects of stressors [9,10]. There is little information on the peculiarities of microalgae AOS action under various abiotic stress conditions and its influence on the composition and amount of biotechnologically valuable compounds accumulated. At the same time, some of these compounds (carotenoids, fatty acids, lipids, vitamins, etc.) perform antioxidant functions in microalgal cells, and an increase in their synthesis is associated with ensuring the protection of microalgae from oxidative damage. Ongoing research has focused mainly on using microalgae containing antioxidant compounds as food and feed ingredients in agriculture, etc., to prevent damage caused by oxidative stress in humans and enteric methane mitigation from ruminant production [11,12,13]. According to scientific publications, research on the growth and simultaneous restructuring of microalgae’s antioxidant defence system and the synthesis of valuable biotechnological compounds is rare [14,15]. Various researchers have conducted studies on individual antioxidant enzymes [16,17,18,19,20]. However, these studies do not provide a comprehensive understanding of the functioning of the antioxidant system of algae. To further improve the strategies for using abiotic stresses to enhance the synthesis of carotenoids, fatty acids, lipids, vitamins, and other compounds that perform an antioxidant function, it is necessary to clarify the behaviour of the AOSs of various types of microalgae.

**Figure 1:**
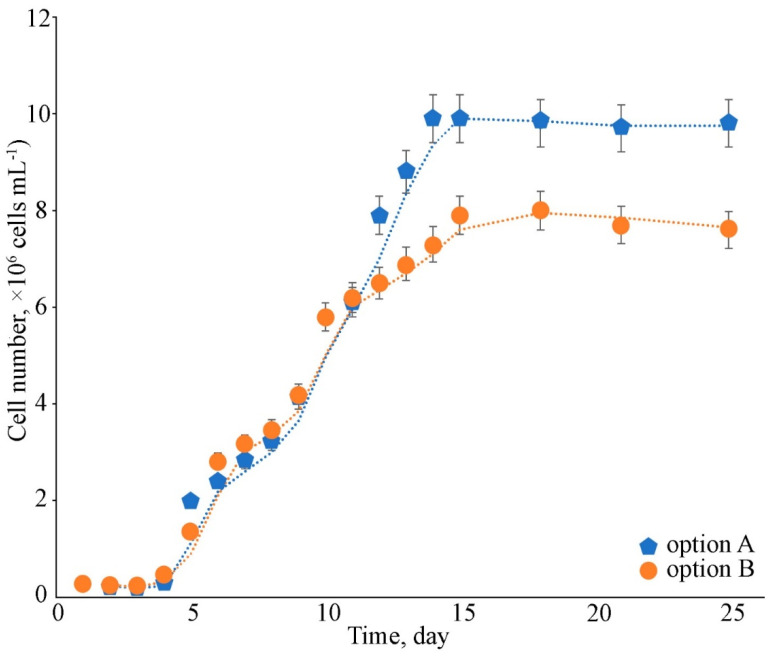
Growth curves of the strain *Cc. oleofaciens* CAMU MZ–Ch4 with different aeration conditions. (Option A) Additional aeration with bubbling speed 0.8 L min^−1^, blue pentagons. (Option B) Limited aeration with bubbling speed 0.1 L min^−1^, orange circles.

When we studied the diversity of soil microalgae in the forest litter of a *Quercus robur* L. plantation in the Samara Forest (Dnipropetrovsk region, Ukraine), the new strain CAMU MZ–Ch4 was isolated in culture and studied [21,22]. Based on the phylogeny of the 18S rRNA and *rbc*L genes, the comparison of ITS2 secondary structures, and light microscopy (Figure 1 at [21]), it was identified as *Chlorococcum oleofaciens* Trainor et H.C. Bold [21]. At the morphological level, the strain was entirely consistent with the revised diagnosis of *Cc. oleofaciens* [23]. At the secondary structure of ITS2, the strain had several differences (one CBC and three *h*CBCs outside the barcode regions) that distinguished it from other known strains of *Cc. oleofaciens*, including the type SAG 213–11 [21].

*Chlorococcum* (*Cc*.) Meneghini is one of the largest genera of the Chlamydomonadales, containing, according to rough estimates, about 50 species [24]. *Chlorococcum* species are widely distributed in various ecosystems [25,26]. *Cc. oleofaciens* is a well-known species of *Chlorococcum* [27] from aquatic and soil biotopes [24], capable of accumulating significant amounts of lipids, up to 59.2% of dry biomass [28]. The high biotechnological value of this species makes studies of its new strain CAMU MZ–Ch4 relevant.

The integral characteristic of the metabolic features of *Cc. oleofaciens* has not yet been identified, and the possibility of activating the synthesis of valuable compounds using abiotic stresses has not been fully assessed. There is information, albeit not always unambiguous, on the results of changes in lipid synthesis and fatty acid composition by *Cc. oleofaciens* strains under the action of different concentrations of iron ions [29], changes in nitrate availability [30,31], and different intensities of the illumination [28]. It should be noted that although stress has been used to promote the biosynthesis of certain compounds, nothing is known about the antioxidant defence system or the activity of its various branches in response to stress in *Cc. oleofaciens*.

Therefore, this work aimed to characterise the content of common lipids, proteins, and pigments in the new strain of *Cc. oleofaciens*, CAMU MZ–Ch4, and to evaluate its antioxidant status in the stress conditions of cultivation created by various aeration conditions (1), nitrogen and phosphorus deficiencies (2), and cultivation duration (3) for the further development of strategies to increase the productivity of this strain in biotechnological production.

## 2. Results

### 2.1. Growth Assessment

The strain *Cc. oleofaciens* CAMU MZ–Ch4 reached the 14th day of culture under the additional aeration (option A) stationary phase (Figure 1).

The growth curve indicates that in the first 4 days, adjustment to the growth conditions occurred. From the 5th day of cultivation, the exponential growth of the strain began and was accompanied by a sharp increase in the number of cells. The specific growth rate in the exponential phase was 0.27 day^−1^. However, in the first 6 days (5th day to 10th), the strain grew more intensely than in the following days of the exponential phase. The specific velocity during these periods was 0.49 day^−1^ and 0.18 day^−1^, respectively. In the limited-aeration version, the stationary phase started on the 16th day, and the specific growth rate was 0.19 day^−1^.

### 2.2. Biochemical Parameters of Strain Cc. oleofaciens CAMU MZ–Ch4 under Different Aeration Conditions

#### 2.2.1. Biomass

**Figure 2:**
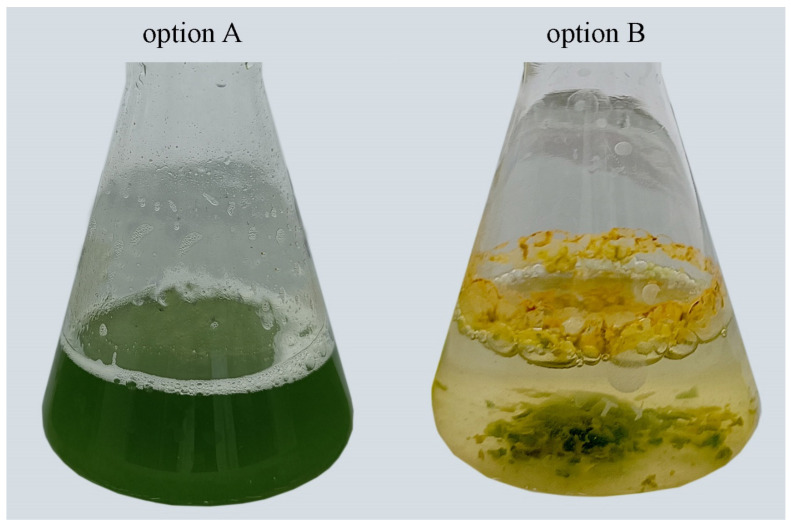
Cultures of the strain *Cc. oleofaciens* CAMU MZ–Ch4 under different aeration conditions. (Option A) Additional aeration. (Option B) Limited aeration.

The different conditions of aeration had a significant impact on biomass accumulation and composition (Figure 2, Table 1). The start of the stationary phase with additional aeration (option A) was matched by a biomass of 1.24 g L^−1^ DW, but the maximum was reached in 18 days with a value of 1.35 g L^−1^ DW. The volumetric biomass productivity on the 18th day was 0.08 g L^−1^ day^−1^. On the 18th day, the limited-aeration version (option B) of biomass reached 1.93 g L^−1^ DW, and the volumetric productivity of biomass was 0.11 g L^−1^ day^−1^. The productivity difference was 30.27%. The pH of the medium after 18 days increased from 6.90 to 10.06 in option A and to 9.92 in option B. Additional aeration contributed to greater alkalinisation of the medium, which may have limited the growth of the strain.

#### 2.2.2. Pigment Composition

The chlorophyll content in option A was 2.86 times higher than that in in option B (Table 1). Additional aeration contributed to higher photosynthetic activity. In the same experiment, cells accumulated large amounts of carotenoids, up to 2.12 ± 0.06 mg g^−1^ DW. Option B exhibited a carotenoid accumulation of 1.63 times less, but even this amount was sufficient to mask the colour of chlorophyll, which was weak (Figure 2b).

#### 2.2.3. Lipid and Protein Contents

The aeration mode affected protein and lipid accumulation in the strain *Cc. oleofaciens* CAMU MZ–Ch4. Additional aeration (option A) was followed by increased biomass protein and decreased lipids (Table 1). The concentration of proteins in the biomass of option A was almost twice as high as that in option B (28.36% and 13.14%), and lipids were half as high (13.74% and 29.07%, respectively).

#### 2.2.4. Fatty- Soluble and Hydrophilic Vitamin Contents

The levels of retinol, ascorbic acid and phenolic compounds in the *Cc. oleofaciens* CAMU MZ–Ch4 biomass differed reliably between the different aerations. In the additional aeration conditions (option A), the contents of ascorbic acid and phenolic compounds were 2.36 times and 1.42 times higher than those in option B. In the limited aeration conditions, these compounds were used to provide a protective function.

The amount of retinol in cell biomass under restricted air access was significantly higher than that for the additional aeration experiment, by 32.11%. This may be due to the higher secondary metabolism rate under option B, as well as to the metabolite consumption under free radicals under option A, which is characteristic under conditions of increased oxygen concentrations and active photosynthesis.

A high content of α-tocopherol characterised both options of strain cultivation and did not differ reliably between them. Thus, the aeration regime had no significant effect on the content of this secondary metabolite. In this case, protection against oxidative damage of the lipids is probably realised at the expense of other components of the antioxidant protection system.

#### 2.2.5. Succinate Dehydrogenase Activity

SDH activity was 3.5 times higher under limited air exchange (option B). The increased enzyme activity indicated an intensive course of anaerobic reactions, which ensure the energy processes of the strain. This may be due to the more intensive methods of cell energy exchange in option B compared to those in option A. Moreover, it is worth noting that catabolism of the protein was observed at the same time. Its content was reduced equivalently to an increase in lipid concentration.

#### 2.2.6. Antioxidant Enzyme Activity

To study the metabolic differences revealed in pigments, lipids, and proteins, the activities of the main enzymes involved in providing antioxidant protection to cells were compared.

Consistent differences in protein-based CAT and SOD activity were observed for different types of experiments (options A and B). The CAT activity in the limited air mixture access variant (option B) was almost twice as high that as in option A. Increased GPx activity was also observed in the strain *Cc. oleofaciens* CAMU MZ–Ch4 in option B. This indicates a more active process of generating and detoxifying hydrogen peroxide and hydroperoxides of lipids formed in cells under these growth conditions.

The increased generation of the superoxide radical and accumulation of a significant amount of organic peroxides and hydrogen peroxide, as well as a corresponding increase in the activity of antioxidant enzymes, testify to the stressed state of the culture of the strain *Cc. oleofaciens* CAMU MZ–Ch4 in option B.

#### 2.2.7. Products of Lipid Peroxidation

According to the results of this study, a reliable reduction in the content of TBA-active products (TBAaP) in the option B was found both in the original homogenate (by 1.63 times) and upon the induction of LPO by the Fenton reaction (Fe^2+^-induced LPO) (by 1.45 times). The decreased TBAaP_in_ content in the biomass of the strain under growth conditions of limited aeration indicates the activation of the cells’ defensive reactions to LPO, which are probably associated with an increase in the content of secondary metabolites.

#### 2.2.8. Antioxidant Activity Coefficient

A study of the strain’s antioxidant activity index (K_AAC_) under cultivation in different aeration conditions showed little difference. A higher value was found in option A, which indicates a greater resistance potential to LPO stress under additional aeration conditions and may be associated with a higher content of low-molecular-weight antioxidants (vitamins A, C, E, P; carotenoids).

### 2.3. Change in Biochemical Parameters and Antioxidant Status of Strain Cc. oleofaciens CAMU MZ–Ch4 in Prolonged Culture

Long-term cultivation was carried out under limited conditions. Therefore, the characteristics of the stationary growth phase strain of option B (Table 1) were used for comparison. In the late stationary growth phase (option C) the biomass concentration was 2.21 g L^−1^. Since the beginning of the stationary stage, its increase was only 12.67%. At the same time, the degradation of pigments was noted, which indicated the activation of cell death processes. The quantities of chlorophyll and carotenoids were only 21.97% and 49.35% of the analogous values at the beginning of the stationary phase. The relatively low carotenoid loss is related to the protective function that carotenoids continue to perform in ageing cells.

The protein quantity decreased to 87.8 ± 10.26 mg g^−1^ DW in the late stationary growth phase, and the lipid content increased to 381.03 ± 3.96 mg g^−1^ DW. Lipids made up 38.10% of DW. Thus, the ageing culture showed a priority accumulation of lipids rather than proteins, corresponding to the late stationary growth phase accompanied by the catabolism of chlorophyll proteins and the accumulation of compounds with cytoprotective activity.

Regarding vitamin content, there was a sharp increase in the concentrations of retinol and α-tocopherol, to 1.4 and 1.6 times those at the beginning of the stationary phase, respectively. This suggests an increasing antioxidant role for retinol and α-tocopherol for metabolism in the ageing cells of the strain. In the late stationary phase, CAT and GPx increased activity, protecting lipids from peroxidation and further decay to TBA-active products. SOD activity decreased, indicating a decrease in aerobic activity. There was also a decrease in SDH activity, which, along with components of the electron transport chain, is a major source of superoxide. The reduction in the activity thereof reduces the load on the strain’s antioxidant protection system.

Decreases in the biomass levels of TBAaP and TBAaP_in_ were also observed, consistent with increased antioxidant resistance in cells. With the increasing age of the culture, the K_AAC_ index became higher, which was consistent with the observed rearrangements in the AOS of the strain.

In general, the observed patterns reveal the peculiarities of the implementation by the strain *Cc. oleofaciens* CAMU MZ–Ch4 of mechanisms for adaptation to long-term cultivation in the conditions of an increasing shortage of nutrients.

### 2.4. Change in Biochemical Parameters and Antioxidant Status of Strain Cc. oleofaciens CAMU MZ–Ch4 in Experiments with Nitrogen and Phosphorus Deficiencies

**Figure 3:**
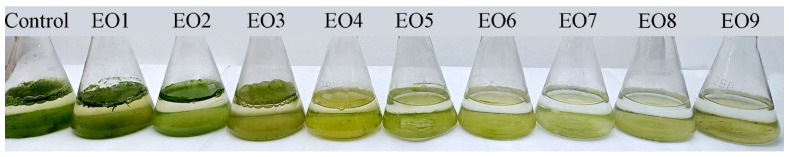
Cultures of the strain *Cc. oleofaciens* CAMU MZ–Ch4 in an experiment with nitrogen and phosphorus deficiencies. EO1–EO9 are experiment options according to Table 2.

Different nitrogen and phosphorus concentrations were created using BBM medium (Table 2). Decreases in nitrogen and phosphorus in the culture medium were accompanied by a progressive decrease in the amount of accumulated biomass (Figure 3; Table 3). This confirms the crucial importance to cell growth of optimal nitrogen and phosphorus supply.

Changes in pigments, proteins, and lipids were not linear with nitrogen and phosphorus concentration decreases. The absolute amounts of nitrogen and phosphorus and their ratio are important.

**Figure 4:**
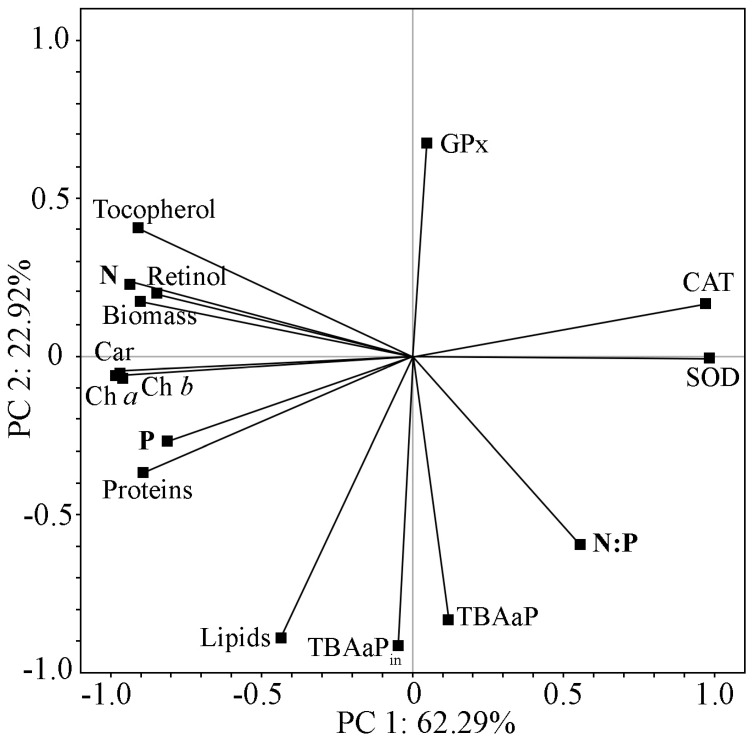
PCA ordination diagram for different parameters measured for the strain *Cc. oleofaciens* CAMU MZ–Ch4 cultivated in media with different nitrogen and phosphorus concentrations and ratios using continuous cultivation. Ch *a*—chlorophyll *a* content; Ch *b*—chlorophyll *b* content; Car—carotenoid content; CAT—catalase activity; GPx—glutathione peroxidase activity; SOD—superoxide dismutase activity; TBAaP—TBA-active products; TBAaP_in_—TBA-active products after induction by the Fenton reaction.

The activity of antioxidant enzymes varied in the different experiment options. There was an increase in CAT and SOD activity with a decrease in nitrogen and phosphorus concentrations in the medium. The change in GPx activity was not unequivocal under the decrease in nitrogen and phosphorus. The TBA content also showed no straightforward dependence on nitrogen and phosphorus. In these cases, the nitrogen and phosphorus ratio in the cultivated medium may have been necessary. PCA was used to determine possible interactions between nitrogen and phosphorus concentrations and their relationships with and effects on the biochemical and antioxidant characteristics of the strain (Figure 4).

The analysis showed that the first two PCs together accounted for nearly 85% of the total variability in the experimental data (Figure 4). PCA 1 primarily reflects the gradient of nitrogen and phosphorus in the medium, and PCA 2 reflects the nitrogen-to-phosphorus ratio. PCA 1 has a strong direct relationship with the characteristics of biomass accumulation, pigment concentration (chlorophyll and carotenoids), and protein, retinol, and α-tocopherol levels. The activities of CAT and SOD show a high but inverse level of communication with PCA 1. PCA 2 shows a direct correlation with lipid accumulation in strain cells and TBA-active products (TBAaP and TBAaP_in_) and a feedback relationship with GPx activity. The intrinsic value of PCA 3 is greater than that of PCA 1, which indicates the usefulness of its isolation and analysis. The concentration of phosphorus in the nutrient medium is most closely related to this factor, and the biochemical characteristics of the strain are retinol and GPx activity. The increase in phosphorus concentration corresponded to a decrease in retinol and an increase in GPx activity. Phosphorus increased phospholipid content, and retinol was used to protect cells from the attack of reactive oxygen species (ROS). Increases in lipid peroxides led to an increase in GPx activity.

### 2.5. Comprehensive Analysis of Biochemical Parameters and Antioxidant Status of Strain Cc. oleofaciens CAMU MZ–Ch4 under Different Culture Conditions

**Figure 5:**
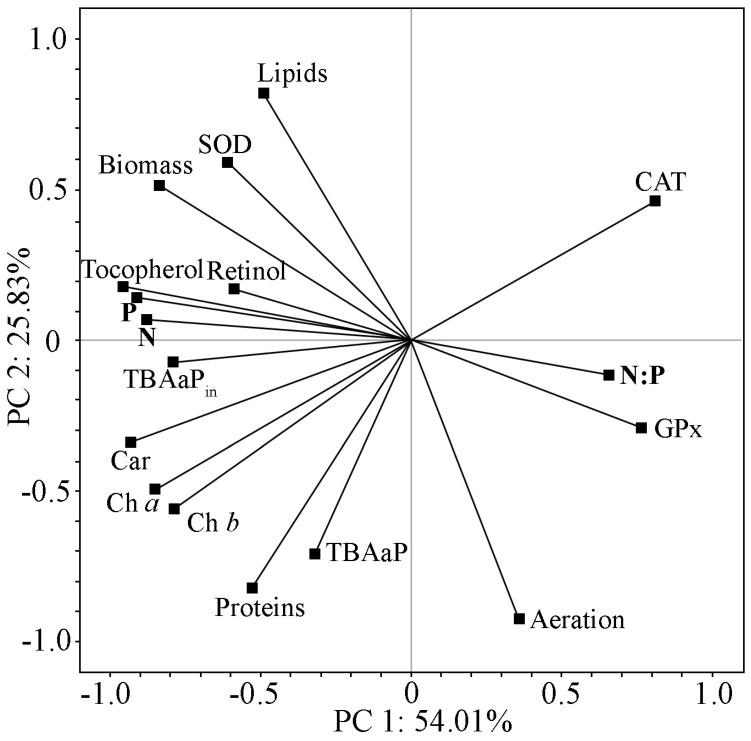
PCA ordination diagram showing the relationship of culture conditions with biochemical and antioxidant characteristics of the strain *Cc. oleofaciens* CAMU MZ–Ch4.

For the complex assessment of biochemical changes in the cells of the strain and the state of its antioxidant system, PCA was used in various experiments. The results of the analysis are presented in Figure 5.

The analysis of the main components for the whole group of variables showed that the first two PCs accounted for 79.84% of the total number of changes noted in the PCA (Figure 5). In this analysis, the amount of nitrogen and phosphorus in the culturing medium and their ratio contributed the most to the structure of PC 1. In this case, a direct relationship between the increase in nitrogen and phosphorus concentration in the nutrient medium and characteristics of the strain such as biomass accumulation, pigment content, and α-tocopherol levels was noted. A nitrogen-to-phosphorus ratio shift towards phosphorus limited biomass accumulation and negatively affected pigment and α-tocopherol content. The SOD activity is directly correlated with PC 1, and the reverse GPx and CAT activities are related. This shows their different roles in the restoration of cell homeostasis under nitrogen and phosphorus deficiency in the nutrient medium and the change in the balance between them. PC 2 reflects the effects of aeration conditions on protein and lipid accumulation characteristics of the strain, representing two alternative metabolic pathways. Aeration conditions also had a significant effect on SOD activity. Upon the induction of LPO, the accumulation of TBA-active products showed a direct relationship with nitrogen and phosphorus availability and coincided with increases in α-tocopherol content. Initially, the accumulation of TBA-active products was more sensitive to aeration conditions. Thus, the limits of the strain’s resistance to LPO are identified when analysing the action of various external factors. In this experiment, nitrogen and phosphorus concentrations are represented by one impact and aeration conditions by the other.

PC 3 describes only 12.58% of the total variance, and since it has an eigenvalue greater than 1, it can be isolated. This component reflects additional interactions between nitrogen concentrations in the medium and retinol.

By combining the results of the two PCs, it can be concluded that under varying aeration conditions, the availability of nitrogen and phosphorus has differing effects on the metabolism of the strain CAMU MZ–Ch4. It also affects the antioxidant defence system, which is rearranged by changing the activity of its various branches.

## 3. Discussion

### 3.1. Growth and Biomass Production

The growth rate of the strain *Cc. oleofaciens* CAMU MZ–Ch4 is strongly influenced by the aeration conditions and the concentrations and ratio of nitrogen and phosphorus in the medium. The maximum growth rate corresponded to 0.27 day^−1^ and was observed in the culture with additional aeration and total nitrogen and phosphorus concentrations of 0.04 g L^−1^ and 0.05 g L^−1^ (control), corresponding to the standard BBM medium.

This growth rate is higher than that previously reported of 0.14–0.19 day^−1^ for the *Cc. oleofaciens* [31]. According to the authors, it became possible to activate algal growth due to the optimisation of the composition of the nutrient medium CFTRI and, especially, the amount of nitrogen and bicarbonate ions in it (NaHCO_3_ was added to the nutrient medium). According to the authors, adding inorganic carbon to the medium contributed to a longer preservation of the culture’s growth potential, including during the stationary phase. Additional aeration in our experiment also contributed to an increase in the growth rate of the strain CAMU MZ–Ch4 and the lengthening of the exponential phase.

Aeration conditions significantly affected biomass production, biochemical composition, and enzyme activity, providing antioxidant protection and energy exchange for the strain *Cc. oleofaciens* CAMU MZ–Ch4. In limited aeration conditions, CAMU MZ–Ch4 was found to exhibit an increased activity of antioxidant enzymes (SOD, CAT, GPx), which indicates a higher level of stress in the cells compared to that under other culture conditions. Moreover, as follows from the PCA, the largest influence of the aeration conditions was on SOD and CAT activity. The expected decrease in biomass with the increase in the stress state of microalgae under limited aeration conditions did not occur. In contrast, the biomass was greater, at 1.93 g L^−1^ (option B), while that under additional aeration was 1.35 g L^−1^ (option A). This was observed by Bouzidi et al. [32] studying *Tetradesmus dissociatus* (P.A.Verses et F.R.Trainor) M.J.Wynne under stress. They concluded that the increase in algae biomass was not due to cell division but to the accumulation of various metabolites within cells. In our experiment, the lipid content of the strain CAMU MZ–Ch4 was significantly increased (more than two times) in cells with limited aeration. Qi et al. [33], studying the growth of *Chlorella vulgaris* Beijerinck in aeration and non-aeration (in sealed glass cans), observed that during aeration, algae lost up to 7.14% of biomass during 12 h of dark cultivation and lipid content increased by 48.25%, while without aeration, the respective rates varied between 0.64 and 1.64% and 0.57 and 8.17%. Thus, the authors noted that biomass losses were significantly higher during oxygenation. Separate studies with a wide range of simultaneously recorded parameters relating to the conditions of cultivation and the morphological, biochemical, and antioxidant characteristics of algae are needed to determine the causes of observed changes in algae metabolism under different aeration conditions.

It is well known that the pH of the medium influences microalgae growth [4]. When microalgae strains are grown, the medium’s pH changes, which is noted in many studies [8,34]. In our experiment, the pH shifted to the alkaline side for 18 days of the experiment (Table 1). In the experimental groups with additional aeration, alkalinisation was more pronounced. Bruno et al. [34] noted that as *Cc. infusionum* (Schrank) Meneghini and other species of green microalgae grew the pH also changed from 6.40 to 7.16–7.34 after fifteen days of cultivation. It has been shown that during the period of crop illumination, the pH rises through photosynthesis and CO_2_ use, but during the dark period, the process moves in the opposite direction through respiration, and the pH decreases again [4]. Intensive photosynthesis in the additional aeration experiment led to a rapid increase in pH, which subsequently negatively affected biomass accumulation. In the experiment with limited aeration, pH increases also occurred, but not so rapidly.

Increased alkalinity levels in algal cultures may also be due to the formation of bicarbonates. It has been reported that the equilibrium between CO_2_ and HCO_3_^−^ depends on pH, and under more acidic conditions, it shifts towards the formation of CO_2_, whereas under more alkaline conditions, it shifts towards the formation of HCO_3_^−^ [35]. The disruption of this balance can occur under the influence of the composition of salts and the content of organic and biogenic substances, metals, etc. The formation of bicarbonates can be affected by the activity of carbonic anhydrase. This enzyme catalyses the reversible hydration reaction of carbon dioxide. Brinkert et al. [35] proved that bicarbonate ions are involved in the mechanisms of control of the redox potential of cells, and they regulate and protect photosystem II. They also demonstrated that changes in the HCO_3_^−^ concentration can affect cellular redox control mechanisms and algal growth. In experiments with *Chlorella vulgaris*, an excess concentration of HCO_3_^−^ at pH 9.5 led to a decrease in algal biomass content since the utilisation of HCO_3_^−^ using the carbonic anhydrase enzyme was energetically unfavourable, according to Li et al. [8].

The study of the effects of nitrogen and phosphorus limitation in the medium showed that the maximum amount of the *Cc. oleofaciens* CAMU MZ–Ch4 biomass was observed at a concentration of 0.0413 g L^−1^ total nitrogen and 0.052 g L^−1^ total phosphorus, corresponding to the standard BBM medium (control). With nitrogen and phosphorus concentrations of 0.00026 g L^−1^ and 0.0002 g L^−1^ (experiment option 9, EO9), the strain culture retained its growth potential, but the dry biomass was minimal, at 0.09 g L^−1^. Some authors point to the possibility of algae growth in a non-sulfuric medium. Bruno et al. [34] reported that the complete absence of nitrogen slowed growth but was not critical for *Cc. infusionum*. In their 15-day experiment, the dry biomass was 0.156 g L^−1^, which was almost two times less than that in experiment with 0.25 g L^−1^ nitrates (total nitrogen 0.041 g L^−1^). Sonkar, Mallick [36] observed the growth of *Chlorella vulgaris* and *Mychonastes homosphaera* (Skuja) Kalina et Puncochárová in a nitrogen-free medium. The biomass concentrations of these species were only 0.18 g L^−1^ DW and 0.20 g L^−1^ DW, lower than those in the experiment with N 11 medium (total nitrogen 0.139 g L^−1^), where the biomass was 1.31 g L^−1^ and 1.39 g L^−1^, respectively.

The increase in nitrogen concentration in the medium, as shown by the literature analysis, does not affect *Cc. oleofaciens* biomass accumulation. Pauline, Achary [31] concluded that the optimal medium (denoted by the authors as AM) for *Cc. oleofaciens* growth should contain NaHCO_3_, NaNO_3_, KNO_3_, and MgSO_4_ at 6.75 g L^−1^, 0.75 g L^−1^, 1.88 g L^−1^, and 0.35 g L^−1^, respectively. On the 15th day of cultivation, a biomass of 1.65 g L^−1^ DW was obtained in the AM medium (total nitrogen 0.3247 g L^−1^), while a biomass of 0.60 g L^−1^ was obtained in the CFTRI medium (total nitrogen 0.247 g L^−1^). The opposite effect was achieved by Adams et al. [37] in experiments to optimise the nutrient medium for several microalgae, including the strain *Cc. oleofaciens* UTEX 105. The maximal biomass of *Cc. oleofaciens* UTEX 105 of 4.3 g L^−1^ DW was accumulated at 4.3 mM nitrate (total nitrogen 0.0558 g L^−1^) in the medium, and at an increase to 11 mM nitrate (total nitrogen 0.1534 g L^−1^), the biomass was only 2.0 g L^−1^. Our experiments have concluded that not only the amount of nitrogen but also that of phosphorus and their ratio have a significant influence on the accumulation of *Cc. oleofaciens* CAMU MZ–Ch4 biomass.

In general, the amount of biomass of *Cc. oleofaciens* CAMU MZ–Ch4 is higher or comparable to that of other biotechnologically valuable species, for example, *Chlorella sorokiniana* Shihira et R.W.Krauss with 0.093 g L^−1^ on BBM medium and 0.688 g L^−1^ on BBM medium with glucose addition [38], *Chlorella vulgaris* with 1.65–2.00 g L^−1^ on 3N BBM medium [39,40] and 1.31 g L^−1^ on N 11 medium [36], *Ettlia oleoabundans* (S.Chantanachat et Bold) J.Komárek with 0.98 g L^−1^ on 3N BBM medium [39] and 1.65 g L^−1^ on BBM medium [41], *Tetradesmus obliquus* (Turpin) M.J.Wynne with 1.1 g L^−1^ on N 11 medium [36] and 0.272–0.659 g L^−1^ on modified 3N BBM medium [42], and *Phaeodactylum tricornutum* Bohlin with 1.74 g L^−1^ on Walne’s medium [39].

The nitrogen-to-phosphorus ratio in the culture medium is one of the most important indicators based on the stoichiometric ratios of key chemical elements in microalgae biomass [4]. These issues have already been the subject of research, but little is known about the sensitivity of various microalgae species to phosphorus deficiency and the nitrogen-to-phosphorus ratio in the medium [43]. The difficulties in understanding the patterns in which nitrogen and phosphorus concentrations and their ratio influence the change in biomass accumulation of the strain *Cc. oleofaciens* CAMU MZ–Ch4 are reflected in the direct and inverse correlation relationships between the variables in the ordinal space formed by PCA 1 and PCA 2. For example, a direct correlation was found between nitrogen and phosphorus concentrations and biomass quantity change, and a negative correlation was observed between the nitrogen-to-phosphorus ratio and biomass accumulation change. Thus, the nitrogen-to-phosphorus ratio in the medium is an important indicator, but more research is needed to determine its optimal values for microalgae growth.

### 3.2. Biochemical Parameters

#### 3.2.1. Lipids, Proteins

Previous work has confirmed that the biomass of different species and strains of microalgae differs in lipid and protein contents [44]. Differences may be due to genetic and environmental characteristics and cultivation conditions [30,45].

The highest lipid content in the biomass of the strain *Cc. oleofaciens* CAMU MZ–Ch4 was obtained in experiments with limited aeration, at 29.1% on the 18th day (option B) and 38.1% on the 74th day (option C). Literature sources provide information about lipid accumulation from 12.0% to 59.18% for *Cc. oleofaciens* [28,37]. A significant increase in lipid content (up to 59.18%) was obtained for the strain *Cc. oleofaciens* f3 with an increase in light intensity up to 400 µmol photons m^−2^ s^−1^ [28]. Regarding the amount of protein stored, *Cc. oleofaciens* MZ–Ch4 showed similar results to *Cc. oleofaciens* SAG 213–11, *Chlorococcum* sp. USMAC 4, and *Chlorococcum* cf. *hypnosporum* D28Z, D37Z, D65Z, D76Z, containing 13.0–35.0% protein, but was inferior to *Cc. amblystomatis* (F.D.Lambert ex N.Wille) N.Correia, J.Varela et Leonel Pereira, which stores protein at 56.67–73.45% [30,45,46,47].

Microalgae have been reported to be able to alter the flow of carbon from proteins or carbohydrates to lipids while limiting nitrogen in the nutrient medium [30]. There is usually a negative correlation between lipid accumulation in microalgal cells and nitrogen concentration in the medium [4]. Such a strategy is considered promising because it reduces the nitrate requirements for large-scale cultivation and contributes to the cost-effectiveness of producing microalgae biomass. Our results and many other reports [37] have shown that periodic microalgae cultures increase in lipid content as the cells move into the ageing stage and nutrient deficiencies increase. In the strain *Cc. oleofaciens* CAMU MZ–Ch4 biomass, the amount of lipids on the 74th day was 38.1% of the dry weight, and on the 18th day, it was 29.1%. The prolonged cultivation and depletion of nutrient stores in the medium hurt protein accumulation. Protein levels decreased from 13.14% to 8.78%. Our data are consistent with the results of Del Río et al. [30], where the greatest accumulation of lipids (about 38.0%) was obtained at nitrate concentrations of 1.0 mM (total nitrogen 0.014 g L^−1^), and an increase in nitrate concentration from 2.0 mM to 10.0 mM (total nitrogen 0.028–0.139 g L^−1^) was accompanied by a decrease in lipid content in the biomass of *Cc. oleofaciens*. These authors observed the opposite effect regarding protein content, where increasing the nitrate concentration from 1.0 mM to 10.0 mM resulted in an increase in protein content from 13.0% to 35.0%.

After analysing our data and that in the literature, it can be concluded that the range of optimal total nitrogen concentration in the medium under the periodic culture mode for the accumulation of lipids by *Cc. oleofaciens* is somewhat higher than that in the standard BBM medium (0.041 g L^−1^). This can be confirmed by the results obtained by Adams et al. [37]. The authors established that the maximum amount of lipids (46.0%) in the biomass *Cc. oleofaciens* UTEX 105 can be obtained with a nitrate concentration of 4.0 mM in the medium (total nitrogen 0.0558 g L^−1^), while higher nitrate concentrations (11.0 mM nitrates; total nitrogen 0.1534 g L^−1^) limited lipid accumulation to 35.0%. Only 12.0% of lipids were detected in a medium with full nitrogen saturation. Pauline, Achary [31] managed to obtain *Cc. oleofaciens* biomass containing 34.0% lipids in the AM medium (total nitrogen 0.3247 g L^−1^). Using CFTRI medium (total nitrogen 0.247 g L^−1^) yielded lower values.

Much less information is available on microalgae’s growth and biochemical characteristics in nutrient media initially containing small amounts of nutrients [48,49]. The cultivation of the strain *Cc. oleofaciens* CAMU MZ–Ch4 in low-nitrogen media with varying phosphorus concentrations did not result in increased lipid or protein contents. The nitrogen-to-phosphorus ratio change in the nutrient medium affected protein and lipid contents (Table 3).

Phosphorus is an essential nutrient for microalgal growth, as it plays a leading role in many metabolic processes [4]. PCA established that changes in nitrogen and phosphorus concentrations in the medium and their ratio have different effects on lipid and protein accumulation. The availability of nitrogen and phosphorus is more important for protein accumulation, and the nitrogen-to-phosphorus ratio in nutrients is more important for lipid accumulation (Figure 3 and Figure 4). Thus, the initially low nitrogen and phosphorus concentrations in the nutrient medium will not stimulate protein and lipid accumulation, and by changing the N/P ratio, cell metabolism can be redirected towards lipid or protein accumulation. Thus, at the N/P ratio of 1.79:1 (EO3) ratio, 9.2% of lipids and 5.68% of proteins were accumulated in the CAMU MZ–Ch4 biomass, and at the N/P ratio of 12.8:1 (EO4) ratio, these values were 13.3% and 10.13%, respectively (Table 1 and Table 3). An increase in protein content in the *Cc. oleofaciens* biomass with an increase in nitrogen concentration and the same phosphorus concentration in the medium was also observed by Del Río et al. [30].

#### 3.2.2. Pigments

Microalgal pigments are greatly valuable for industrial use because of their bioactive potential [50]. Among green microalgae, *Dunaliella salina* (Dunal) Teodoresco and *Haematococcus lacustris* (Girod-Chantrans) Rostafinski [51] are considered valuable sources of carotenoids. Recently, it has been reported that the biomass of other green microalgae is rich in carotenoids. These include *Chromochloris zofingiensis* (Dönz) Fucíková et L.A.Lewis, *Coelastrella striolata* Chodat, *Scenedesmus* Meyen species, and some *Chlorococcum* [44,52]. It is known that β-carotene, astaxanthin, astaxanthin esters, canthaxanthin, lutein, and other carotenoids may accumulate in *Chlorococcum* cells [53].

High levels of photosynthetic pigments in the strain *Cc. oleofaciens* CAMU MZ–Ch4 were found in the experiment with additional aeration (Table 1). The total chlorophyll content in the early stationary phase reached 6.37 mg g^−1^ DW, and the carotenoids reached 6.35 mg g^−1^ DW. The Ch *a* content of the studied strain was lower than that known for the strain *Cc. infusionum* TISTR 8461, with 8.45 mg g^−1^ DW [54]. The carotenoid content of the strain CAMU MZ–Ch4 is higher than that of *Cc. infusionum* TISTR 8461, with 4.14 mg g^−1^ DW [54], and lower than those of several strains of *Chlorococcum*, ranging from 12.26 to 21.02 mg g^−1^ DW [45], as well as *Chlorococcum* sp. MC1, with 15.10 mg g^−1^ DW [13].

In nitrogen- and phosphorus-deficient experiments (Table 3), a decrease in chlorophyll was observed, indicating a limited photosynthetic capacity under stress conditions. Decreases in photosystem II efficiency due to decreased photosynthetic pigments have been noted previously in *Chlorella sorokiniana* under nitrogen starvation [16]. The authors also reported an increase in carotenoids relative to chlorophyll. In our experiment, the ratio of Ch *a*/Car was 1:0.43 due to the accumulation of secondary carotenoids, and with nitrogen and phosphorus deficiency, it increased even more. It has been reported that under stressful conditions (lack of nutrients, bright lighting, salt stress, etc.), the photosynthetic system cannot efficiently use the energy from the absorbed light [55]. When microalgal cells receive a high-level recovery signal, a mechanism is triggered within the cells to disperse the accumulated electrons. This mechanism involves the synthesis of antioxidant carotenoids [55]. The combination of nitrogen starvation with varying amounts of phosphorus in the medium resulted in different changes in chlorophyll and carotenoid contents (Table 3). PCA found that increases in nitrogen and phosphorus contributed to increases in carotenoids and chlorophyll, and for the latter, increased aeration was also important (Figure 3 and Figure 4).

#### 3.2.3. Vitamins

Microalgae have a high potential as a source of various vitamins. They can synthesise vitamins in quantities greater than or comparable to the release of plant analogues [56].

Such species as *Chlamydomonas pyrenoidosa* Schiller, *Chlamydomonas reinhardtii* P.A.Dangeard, *Chlorella sorokiniana*, *Chlorella vulgaris*, Desmodesmus *communis* (E.Hegewald) E.Hegewald, *Dunaliella tertiolecta* Butcher, *Nannochloropsis* oculata (Droop) D.J.Hibberd *Tetradesmus obliquus*, *Tetraselmis suecica* (Kylin) Butcher, and some others are known as preferred producers of vitamins A, C, E. [44,56]. Therefore, their indicators were prioritised to assess the prospects of the strain *Cc. oleofaciens* CAMU MZ–Ch4 as a producer of these vitamins.

The strain *Cc. oleofaciens* CAMU MZ–Ch4 accumulated α-tocopherol in the range of 0.43–0.49 mg g^−1^ DW, retinol in the amount of 0.08–0.16 mg g^−1^ DW, and ascorbic acid and phenolic compounds at 0.08–0.19 mg g^−1^ DW and 0.23–0.32 mg g^−1^ DW, respectively.

The α-tocopherol content in the biomass of CAMU MZ–Ch4 was comparable to that in *Chlorella vulgaris* (0.4–0.6 mg g^−1^ DW), *Dunaliella tertiolecta* (0.5 mg g^−1^ DW), and *Tetraselmis suecica* (0.6 mg g^−1^ DW) [57,58]. According to Del Mondo et al. [11], retinol content changes in microalgae biomass from 0.01 mg g^−1^ to 4.80 mg g^−1^ DW, and in the strain *Cc. oleofaciens* CAMU MZ–Ch4, it was higher than in *Nannochloropsis* D.J.Hibberd species (0.05–0.08 mg g^−1^ DW), and *Chlorella* Beyerinck [Beijerinck] (0.01–0.65 mg g^−1^ DW) and was comparable to that in *Chlamydomonas* Ehrenberg (0.11–0.13 mg g^−1^ DW).

The ascorbic acid content in the CAMU MZ–Ch4 biomass was lower than, for example, that of *Nannochloropsis oculata* (0.75% DW) and *Chaetoceros gracilis* Pantocsek (1.62% DW) [59], but from the point of view, for example, of feeding animals in aquaculture, which may require 0.003–0.02% ascorbic acid in their diet, it is reasonably sufficient.

Vitamin content depends on nutrient availability, which has been well confirmed by PCA (Figure 4 and Figure 5). The literature has also reported that nitrogen restriction in the medium is usually accompanied by an increase in tocopherol content [60]. Phosphorus restriction has also been shown to lead to an increase in the concentration of tocopherols. The concentration of α-tocopherol in the experiment increased from 0.428 mg g^−1^ in the early stationary phase (option B) to 0.684 mg g^−1^ DW in the late stationary phase (option C). This is consistent with the results of Durmaz [61] for *Nannochloropsis oculata*, which contained 0.5 mg g−1 α-tocopherol at the end of the exponential phase and 2.33 mg g^−1^ DW in the late stationary phase. Growth in the initial nutrient-poor media (Table 3) was accompanied by limited synthesis of α-tocopherol and retinol. This reduces the stress resistance of cells, and in this case, protection against ROS is provided by other components of the antioxidant system, for example, by carotenoids and antioxidant enzymes.

Singh et al. [62], studying *Monoraphidium* sp., reported that with nitrogen deficiency, the total amount of tocopherols increased due to δ-tocopherol, while the content of α-tocopherol decreased. They also noted that under stress in *Monoraphidium* sp. cells, δ-tocopherol levels fell after day 6, and this may be because tocopherols degrade and are processed as ROS concentrations increase in chloroplasts. It is known that tochs (α, β, γ, δ), the biological activity of which is not the same [63]. The composition of the tocopherol isoforms can also play a role in the formation of protective reactions under stress.

An extensive group of phenolic compounds is involved in various algal physiological processes, including antioxidant protection [64]. The number of phenolic compounds in algae varies widely from 0.2 mg g^−1^ to 39.9 mg g^−1^ and 58.15 mg of pyrocatechol equivalents [65,66]. The strain *Cc. oleofaciens* CAMU MZ–Ch4 exhibited phenolic compound contents of 0.23–0.32 mg g^−1^ DW in ethanol extract in the early stationary phase. According to other reports, the content of phenolic compounds in *Chlorococcum* sp. 53 biomass was higher and amounted to 2.9 mg g^−1^ DW in the aqueous extract and 3.7 mg g^−1^ DW in the ethanol extract [67]. Similar phenolic compounds were found in *Chlorella vulgaris* in methanol extract, with contents ranging from 0.75 to 2.21 mg g^−1^ DW [68] and in *Chaetoceros curvisetus* Cleve, with 0.44 mg g^−1^ DW in acetone extract and 0.51 mg g^−1^ DW in the methanolic extract [69]. The analysis of our data and other reports shows that the number of phenolic compounds varies among microalgae and depends on growth conditions. Differences in phenolic compounds may also be related to the different solubilities of individual phenolic compounds synthesised by algae in the solvents used in experiments [70].

#### 3.2.4. Antioxidant Defence System Enzymes

When environmental stressors affect algae, the generation of ROS in cells increases. The accumulation of ROS can lead to severe disturbances in the biochemical mechanisms of the cells, damage to various components of the cells, and the peroxidation of lipid membranes.

*Chlorococcum* species are widespread in various soil and aquatic ecosystems and have a wide range of adaptability to different habitats. The ecological range of natural habitats of *Cc. oleofaciens* is also vast. It is noted in the soils of various forest, steppe, and meadow ecosystems; tundra communities; marine and dune communities; etc. [21,22,25,71,72,73]. Extremophile isolates are known from soils, volcanic emissions, water reservoirs for storing palm oil, mineralised waters (14‰) of the Jara River, etc. [23,72,74]. Thus, the overall case study for adaptations of different isolates of *Cc. oleofaciens* is quite broad and presumably covers both physiological and genetic changes. The diversity of lipid, protein, and other metabolite contents observed by various authors in different strains of *Cc. oleofaciens* is evidence to show their individual metabolic phenotypes. The actions of stressors trigger a series of metabolic changes that can be different in strength and consequence and depend on the metabolic capabilities of a particular strain and its individual case adaptation. This hypothesis is consistent with the conceptual understanding of stress as a disorder of homeostasis due to stress and stress response as a change in cellular metabolism during acclimatisation and homeostasis repair, as outlined by Borowitzka [9].

When stress acts on algal cells, AOS defence mechanisms are triggered. The AOS includes low-molecular-weight antioxidants (α-tocopherol, ascorbic acid, carotenoids, etc.) and a range of enzymes (catalase, peroxidases, superoxide dismutase, etc.) [75]. An important fact is that many target products of biotechnological production, such as carotenoids, lipids, vitamins, etc., are components of microalgal AOSs or act as substrates for peroxide oxidation and oxidation modification. The success of using stress to stimulate the accumulation of these compounds is directly related to the discovery of the mechanisms of operation of microalgal AOSs. Obviously, in this case, it is essential to separate the moments of stimulation of the synthesis of compounds with antioxidant properties and their expenditure in neutralising/extinguishing ROS. It has been suggested that manipulating intracellular ROS by inducing an intracellular redox state, either by nutrition or by inducing a specific cellular metabolism, could be a practical approach to increasing secondary carotenoid production [32].

The present experiments revealed a change in the composition of metabolites, including those with antioxidant properties, as well as enzymes and antioxidants, occurring under different conditions of aeration, during long-term cultivation with the exhaustion of nutrients in the medium, and during growth in media with an initial low concentration of nitrogen and phosphorus.

When additional aeration was used, the cells contained more ascorbic acid, carotenoids, and phenolic compounds. Under limited aeration, the protective function was shifted to antioxidant enzymes (CAT, SOD, GPx) and lipids containing fatty acids, including unsaturated acids. Their role in ROS quenching is well documented in the literature [76,77]. There was also a significant decrease in SDH activity under additional aeration, which may be an alternative mechanism for increasing antioxidant resistance by reducing the FAD-coupled generation of ROS by the enzyme [78,79]. In conditions of limited aeration, increased SDH activity may play an essential role in maintaining the redox balance of cells, as the conversion of substrates into the Krebs cycle is an aerobic process. The higher activity of this enzyme compensates for the lower oxygen concentration, and this is probably a typical pattern of the mitochondrial regulation of SDH activity and requires further study to understand the features of the metabolome (broadly understood, including lipids, etc.) of microalgae and develop strategies to control energy exchange in cells.

Nutrient depletion during long-term culture was accompanied by a decrease in the carotenoid count in cells and a significant accumulation of α-tocopherol.

Increases in nitrogen and phosphorus increased CAT and SOD activity, and decreased carotenoids, retinol, and α-tocopherol characterised depleted media. The data we obtained on the change in SOD activity in the strain *Cc. oleofaciens* CAMU MZ–Ch4 is confirmed by the results of earlier studies [17], in which an increase in SOD and CAT activity in *Tetradesmus dimorphus* (Turpin) M.J.Wynne grown in conditions of nitrogen starvation was noted.

Bouzidi et al. [32], studying *Tetradesmus dissociatus*, noted that the biosynthesis of carotenoids and lipids was altered by the inhibition of antioxidant enzymes such as SOD, CAT, GPx, and ascorbate peroxidase. The highest concentrations of carotenoids and lipids occurred with CAT inhibition. This is consistent with our observations. In lower-CAT-activity experiments, carotenoids and lipids were higher. Thus, in the strain *Cc. oleofaciens* CAMU MZ–Ch4, a cell protection function was provided by different components of the antioxidant protection system in different stress situations.

To assess the antioxidant status of microalgae, various coefficients are often used, for example, 2,2-diphenyl-1-picryl-hydrazyl radical scavenging activity (DPPH), 2,2′-azino-bis(3-ethylbenzothiazoline-6-sulfonic acid) radical scavenging activity (ABTS), ferrous ion-chelating ability, the antioxidant activity coefficient (KAAC), etc. [80,81,82,83,84]. These coefficients are integral indicators of the antioxidant defence system state and reflect the cells’ overall ability to neutralise the active forms of oxygen and peroxides. Based on the K_AAC_ values, it can be stated that the antioxidant protection system of *Cc. oleofaciens* CAMU MZ–Ch4 is resistant to nitrogen and phosphorus starvation. The prolonged cultivation of the strain is accompanied by an increase in the overall antioxidant status of cells, which is probably due to the accumulation of retinol and α-tocopherol.

## 4. Material and Methods

### 4.1. Microalgal Material

The strain *Cc. oleofaciens* MZ–Ch4 was isolated from forest litter in the oakwood of the Samara Forest (N 48°39′30.44″, E 35°39′2.17″). The strain was deposited in the Algae Collection of Molecular Systematics of Aquatic Plants at the K.A. Timiryazev Institute of Plant Physiology RAS and the Collection of Algae at Melitopol State University CAMU (WDCM1158) as perpetually transferred pure cultures. The strain is stored at 15.0 ± 2.0 °C in vials illuminated by white diodes with a light intensity of 120 lx and a light mode of 16:8 h (light/dark) in BBM medium [85]. A culture in the exponential phase was used for the experiments. For this purpose, 10.0 mL *Cc. oleofaciens* was inoculated in 150.0 mL of fresh BBM. After 5 days of growth, this culture was used for introduction into experimental media.

### 4.2. Experimental Design

To assess the growth and biochemical characteristics of the strain, it was grown in Erlenmeyer flasks with a volume of 250.0 mL and 150.0 mL of BBM at 23.0 ± 2.0 °C. The initial cell concentration was 2.89 × 10^5^ cells mL^−1^. Cell concentrations were measured with a TC20 Automated Cell Counter (Bio-Rad Laboratories, Hercules, CA, USA). The light intensity was 5000 lx (70.0 µmol photons s^−1^ m^−1^) with a colour temperature of 4000 K, and the illumination mode was 16:8 (light/dark). The light intensity and colour temperature were measured using a Sekonic C-800 spectrometer (Sekonic Corporation, Tokyo, Japan). The crops were grown in stagnant mode without shaking.

The biochemical parameters were studied during the early and late stationary phases. 

To study the effects of aeration, two modes (options A and B) of air supply were set using the Hailea ACO–308 aquarium compressor (Hailea, Chaozhou, China). Air was supplied through a glass tube with a 4 mm diameter. During the additional aeration, bubbling was carried out at a speed of 0.8 L min^−1^ (option A) and during limited aeration at 0.1 L min^−1^ (option B). The pH value during cultivation was not corrected and was measured at the end of the experiment to estimate the change in pH under different aeration modes. Also, a culture with air bubbling at a speed of 0.1 L min^−1^ was used to assess changes in the strain’s biochemical characteristics and antioxidant status during long-term cultivation. The strain was grown in this experiment until the late stationary phase (74 days, option C). The effect of different nitrogen and phosphorus concentrations was studied in the early stationary phase with air bubbling at a speed of 0.1 L min^−1^.

Nutrient concentrations for the experiments were calculated based on previously proposed recommendations for standardising microalgae cultivation conditions [7]. Samples with the standard nitrogen and phosphorus concentrations for the medium were used as controls (Table 2). The total nitrogen and phosphorus concentrations were calculated as NO_3_–N and PO_4_–P. Cultivation continued until the early stationary phase.

Cultures for all experiments (Options A, B, C, EO1–EO9) were carried out in three repetitions. Before determining the biochemical characteristics of the algal cells in each variant, the algal biomass was washed from the medium with distilled water and deposited by centrifugation (4000 rpm, 10.0 min), and the liquid above the sludge was removed. This process was repeated three times.

### 4.3. Growth Assessment

The growth was followed for 25 days. For the first 15 days, growth was measured daily, and it was measured every 3 days after that. The growth of microalgae was estimated by measuring the dry weight (DW). The measured dry weight is expressed in g L^−1^.

The specific growth rate µ (per day) was calculated by using the following equation:

µ (day^−1^) = ln (X_1_ − X_0_) (t_1_ − t_0_)^−1^,

where X_1_ and X_0_ are the final and initial biomass concentrations (g L^−1^) on days t_1_ and t_0_, respectively [86].

The biomass productivity (BP, g L^−1^ day^−1^) was estimated using the following equation, where X (g L^−1^) is the concentration of the biomass at the end of the cultivation time t and X_0_ (g L^−1^) is the concentration of the biomass at the beginning of the cultivation [87]:

BP (g L^−1^ day^−1^) = (X − X_0_) t^−1^.

### 4.4. Biochemical Parameters

#### 4.4.1. Measurement of Chlorophyll Content

The determination of chlorophyll content was performed using spectrophotometry [88]. The extraction of pigments from microalgae was carried out with 100.0% acetone. For this purpose, 10.0 mg of biomass separated from the medium by centrifugation (10 min; 3000 rpm; 530× *g*) was subjected to triplicate freezing–defrosting to destroy the cell envelopes. After that, 4.0 mL of acetone was added to create a ratio of biomaterial to extractant of 1:80 (*w*:*v*), and the biomass was additionally homogenised by rubbing with quartz sand. The resulting mixture was hermetically sealed and left for 24 h at a temperature of 25.0 °C in a dark place for the complete extraction of pigments. Further, the acetone extract was separated from the sediment by centrifugation (10 min; 10,000 rpm; 6000× *g*; 4.0 °C). The absorption intensity of the pigment extract was analysed on a Ulab 102 spectrophotometer (Ulab, China) at wavelengths of 662 and 645 nm, corresponding to the absorption maximums for Chl *a* and *b*. The chlorophyll content was expressed in mg g^−1^ of dry biomass, and calculations were carried out according to the following formulas [88]:

Chl *_a_* = 11.85 E_662_ – 2.35 E_645_

Chl *_b_* = 18.61 E_645_ − 3.96 E_662_

#### 4.4.2. Measurement of Carotenoid Content

Carotenoid levels were determined using spectrophotometry [87]. The method involved extracting the sum of the pigments from the biomass using 100.0% acetone, with further measurement of the maximum absorption of the carotenoid fraction at 470 nm and additional consideration of the effect of the absorption of chlorophyll *a* and *b*. The analysis of the extract was carried out on the Ulab 102 spectrophotometer (Ulab, Nanjing, China). The concentrations were expressed in mg g^−1^ of dry biomass and calculated using the following formula [88]:

Car = (1000 E_470_ − 2.270 Chl *a* − 81.4 Chl *b*) 227^−1^

Pigment extract was obtained by adding 4.0 mL of 100.0% acetone to 5.0 mg of biomass separated from the medium by centrifugation (10 min; 3000 rpm; 530× *g*). The biomass was previously subjected to triplicate freezing–defrosting and 24 h extraction at a temperature of 25.0 °C in a dark place. The sediment was then separated by centrifugation (10 min; 10,000 rpm; 6000× *g*; 4.0 °C).

#### 4.4.3. Vitamin A (Retinol) and E (α-Tocopherol) Content Measurement 

Retinol and α-tocopherol were analysed with thin-layer chromatography. Biomass (30.0 mg) was separated from the medium (10 min; 3000 rpm; 530× *g*), and 10.0 mg of ascorbic acid and 1.0 mL of a 10.0% solution of 0.5 N KOH in ethanol (Sigma-Aldrich, St. Louis, MO, USA) were introduced into the glass vial. The resulting mixture was then washed for 30 min at a temperature of 90.0 °C. The resultant hydrolysate was cooled to room temperature, and the hydrophobic fraction was extracted by multi-stage extraction (4 times by 2.0; 1.0; 1.0; 1.0 mL) using hexane, pre-adding 1.0 mL distilled water to the hydrolysate. After settling and separating the hexane and aqueous ethanol phases, the hexane phase was decanted and transferred to a dry vial. The combined hexane extract was washed from KOH with distilled water and subjected to a wash-water-neutral reaction on a universal indicator paper. Then, the extract was dried by freezing water at −18.0 °C. The resulting dry hexane extract was evaporated in a vacuum at 55.0–60.0 °C. The dry residue was dissolved in 100.0 µL benzene (PanReac AppliChem, Barcelona, Spain). To separate vitamins onto a high-performance thin-layer chromatography (HPTLC) plate, (Merck Millipore, Sigma-Aldrich, Supelco) silica gel 60 was applied to 10.0 µL of the resulting solution in three agility, and Sigma-Aldrich (USA) solutions were applied in parallel to determine the position of vitamin stains on the plate. The mobile phase was chloroform (Sigma-Aldrich, USA) for α-tocopherol [89] and acetone–petroleum ether (18:82; *v*:*v*) for retinol [90]. To colour the substances on a TLC-plate, 1.0% phosphomolybdic acid (LenReaktiv, Saint Petersburg, Russia) was used in 96.0% ethanol acidified by HCl (LenReaktiv, Russia). After the separation of the substances, the plate was coloured by immersing it in a dye solution for 10.0 s with further heating of the plate at 100.0 °C for 1 min. Retinol and α-tocopherol appeared as blue spots on a yellow background, further bleached by ageing in a vapor-saturated ammonia chamber for 20 s. The plates were scanned with a Canon MF3010 (Canon, Tokyo, Japan) scanner at a resolution of 300 dpi and analysed in Sorbfil TLC Videodensitometr ver. 2.3.0 software (Sorbfil, Krasnodar, Russia). The concentration was determined by comparing the product height and areas of peaks with similar calibration charts constructed using standard retinol (Sigma-Aldrich, USA) and α-tocopherol solutions (Supelco, Darmstadt, Germany). The standard solution concentration is 1 µg µL^−1^.

#### 4.4.4. Vitamin C (Ascorbic Acid) and P (Phenolic Compounds) Content Measurement

The phenolic compound content of the samples was determined according to the Folin–Chocalteu colourimetric method according to a protocol described by Vazquez et al. [91]. To achieve this, 30.0 mg of dry microalgae biomass was placed in a sealed vial with a lid, and 0.5 mL of 50.0% methanol (LenReaktiv, Russia) was added containing 1.0% of trichloracetic acid (TCA). To ensure complete extraction of phenolic compounds and the precipitation of protein molecules containing aromatic amino acids, the vial was blown with argon, sealed with a lid, and extracted at 80.0 °C for 20 min. The vials were cooled and centrifuged for 15 min at 4000 rpm to precipitate insoluble components. A 0.20 mL aliquot was then placed in a Falcon vial, and 100.0 µL of Folin–Chocalteu phenolic reagent (Scharlab, Barcelona, Spain) and 1.0 mL of Na_2_CO_3_ solution (20.0%, *w*:*v*) were added. The vials were placed in a dark place at room temperature for 30 min. The optical density of the solution was then measured at 760 nm using the ULab 102 spectrophotometer (ULab, China). The total phenolic compound content was quantified with a calibration curve obtained using a standard solution of rutin (Rut) (Merck, Darmstadt, Germany) in a range from 1.0 to 20.0 mg L^−1^ (R^2^ = 0.999) and the mg equivalent of rutin at 1.0 g DW (mg Rut g^−1^).

The method of Kampfenkel et al. [92] was used to determine the content of ascorbic acid. For this purpose, 30.0 mg of microalgae biomass was used, previously subject to freezing and defrosting in a centrifugal plastic vial with a lid. Then, 1.0 mL of 10.0% trichloroacetic acid (LenReaktiv, Russia) was added to the biomass and homogenised for 10 min at 4.0 °C. Further, 800.0 µL of 42.0% H_3_PO_4_, 800.0 µL of 4.0% 2.2′-dipyridyl (Sigma-Aldrich, USA) (in 70.0% ethanol), and 400.0 µL of 3.0% FeCl_3_ (in H_2_O) were introduced into the incubation mixture. The mixture was incubated at 42.0 °C for 45 min and then centrifuged (5 min; 5000 rpm; 4.0 °C). The optical density of the solution was then measured using a spectrophotometer at 525 nm. A calibration chart was used to quantify the optical density (µg g^−1^ DW). The standard was a calibration solution made with crystalline ascorbic acid (Supelco, Germany).

#### 4.4.5. Succinate Dehydrogenase Activity Measurement

Succinate dehydrogenase (SDH) activity was determined using the method of Munujos et al. [93]. The method is based on the ability of the enzyme to transfer reduced equivalents from the substrate to the artificial acceptor 3-(4-Iodophenyl)-2-(4-nitrophenyl)-5-phenyl-2H-tetrazol-3-ium chloride. The result is a coloured compound with maximum absorption at 500 nm. The volume of the reaction medium and the sequence of application of the reagents and the biomaterial were determined by the standard kit protocol for assessing succinate dehydrogenase activity (Merck, Germany). The following steps were taken to prepare the supernatant for analysis: A microalgae biomass of 0.1 g was separated from the nutrient medium by centrifugation (10 min; 3000 rpm; 530× *g*) and washed with distilled water. It was then homogenised in 0.9 mL of phosphate buffer (0.1 M; pH 7.8) during cooling (4.0 °C). The supernatant was separated from the sediment for further analysis by centrifugation (10 min; 10,000 rpm; 6000× *g*; 4.0 °C).

#### 4.4.6. Antioxidant Enzyme Activity Measurement

Homogenate was prepared to determine the activity of antioxidant enzymes. Biomass was separated from 0.1 g of medium by centrifugation (10 min; 3000 rpm; 530× *g*) and homogenised with 0.9 mL of phosphate buffer (0.1 M; pH 7.5; 4.0 °C). Then, 0.25 mL of 96.0% ethanol (LenReaktiv, Russia) and 0.15 mL of chloroform (LenReaktiv, Russia) were added to the resulting homogenate and mixed for 15 min at a temperature of 0.0–2.0 °C to separate lipophilic substances. For further analysis, an upper water–ethanol extract of enzymes (colourless) was used, which was separated from chloroform (lower, green) by centrifugation and decantation. Glutathione peroxidase activity was determined in a homogenate not exposed to ethanol or chloroform. The supernatant was produced by centrifuging (10 min; 10,000 rpm; 6000× *g*; 4.0 °C) the homogenate in phosphate buffer with further decanting.

Glutathione peroxidase (GPx) activity (EC 1.11.1.9) was determined using the method of Razygraev, Arutiunian [94], which is based on the enzyme’s ability to convert glutathione in the process of peroxide reduction with the further formation of coloured conjugates of residual glutathione with Ellman’s reagent (5,5′-dithiobis-(2-nitrobenzoic acid)), which has a maximum absorption at a wavelength of 412 nm.

Catalase (CAT) activity (EC 1.11.1.6) was determined using the Góth [95] method. This method is based on the enzyme’s ability to convert H_2_O_2_ upon interaction with ammonium molybdate into a sustainably coloured complex with a maximum absorption of 410 nm. The activity was calculated from the residual H_2_O_2_ content of the final solution.

Superoxide dismutase (SOD) activity (EC 1.15.1.1) was determined using the Fried [96] method. The method is based on the ability of the enzyme to inhibit the reduction of nitroblue tetrazolium dye under the conditions of generation of superoxide anion radical in the NADH oxidase system.

The preparation of microalgae biomass for measuring antioxidant enzyme activity was carried out according to the protocol described by Yakoviichuk et al. [84].

#### 4.4.7. Measurement of TBA-Active Product Content

The content of secondary lipid degradation products (TBA-active products, TBAaP) during lipid peroxide oxidation (LPO) was determined using the method of Zeb, Ullah [97]. The method is based on the ability of TBA-active products to form a coloured complex (maximum absorption of 532 nm) with 2-thiobarbituric acid in an acidic medium when heated. The content of the products was determined in the initial homogenate (TBAaP) and during the induction of LPO by Fe^2+^ ions (TBAaP_in_). The extract of TBA-active products was prepared by the homogenisation of 50.0 mg of biomass previously separated from the medium by centrifugation (10 min; 3000 rpm; 530× *g*) and decantation of the supernatant liquid. Further, 0.45 mL of 1.2% KCl (LenReaktiv, Russia) solution was added to the precipitate, and the biomass was homogenised. The homogenate was then centrifuged (10 min; 10,000 rpm; 6000× *g*; 4.0 °C), and the supernatant was used for further research.

#### 4.4.8. Antioxidant Activity Coefficient Calculation

The antioxidant activity coefficient (K_AAC_) was used as a marker of the general state of the AOS. The calculation was performed by correlating TBAaP with TBAaP_in_ [84]:

K_AAC_ = TBAaP TBAaP_in_^−1^

#### 4.4.9. Lipid Content Measurement

The determination of the total lipid content was carried out using the gravimetric method. To achieve this, 50.0 mg of separated biomass was transferred to a pre-weighted sample bottle with a lid. The sample was frozen and defrosted, dried in a vacuum at 60.0 °C until constant mass was obtained, and weighed to determine the residual mass. The dry residue was extracted five times with a mixture of hexane-propane-2-ol in a ratio of 3:2 (*v*/*v*) [98] in volumes of 2.0, 1.0, 1.0, 0.5, and 0.5 mL. Before extraction, 1.0 mL of 0.9% NaCl (LenReaktiv, Russia) solution was added to the dry residue to better separate the lipophilic and hydrophilic phases. To drain the extract, the top hexane layer was transferred by decantation to a 25.0 mL chemical glass, into which the waterless Na_2_SO_4_ (LenReaktiv, Russia) layer of 1.0 mm was previously poured. Then, the extract was poured into a pre-weighted sample bottle, and a glass with precipitate was washed twice with hexane (LenReaktiv, Russia). The extracts were combined and evaporated in a vacuum at 60.0 °C. The dry lipid fraction was weighed and expressed in mg g^−1^ dry mass.

#### 4.4.10. Measurement of Protein Content

The hydrophilic protein content was measured to calculate the activity of enzymes. For this purpose, the protocol of Olson [99], which is based on the bicinchoninic method, was used. To obtain the protein extract of 10.0 mg of biomass separated by centrifugation from the BBM medium, three rounds of freezing–defrosting and homogenisation in 1.0 mL of ethanol (LenReaktiv, Russia) were carried out to remove lipophilic substances. The resultant homogenate was centrifuged (10 min; 10,000 rpm; 6000× *g*; 4.0 °C), after which the supernatant liquid was decanted, and the residue was resuspended in 1.0 mL of phosphate buffer (0.1 M; pH 7.5) containing 0.5% sodium dodecyl sulphate (LenReaktiv, Russia) by mass. The mixture was left in a hermetically sealed vial for 12.0 h at 25.0 °C for complete protein extraction. The homogenate was re-centrifuged (10 min; 3000 rpm; 530× *g*), and the supernatant was used to analyse protein content. The calibration curve was constructed according to the standard solution of bovine serum albumin (Thermo Scientific, Waltham, MA, USA).

### 4.5. Data Analysis

All measurements were carried out in three repetitions. The graphs show the average values and errors of the average. Statistical analysis was carried out using XLSTAT 2018 software (New York, NY, USA). Statistics were obtained in Microsoft Excel ver. 1903 software using single-factor dispersion analysis (ANOVA). The reliability of the differences between the indicators was calculated using the Tukey–Kramer posterior test. Differences at *p* ≤ 0.05 were considered reliable. Calculations and plotting were performed using Statistica ver. 12.0 software. 

A principal component analysis (PCA) established the relationship between the measured parameters. PCA is widely used in cases where algal growth is affected by many physical and chemical factors and its mechanism of action is complex to describe using traditional statistical approaches. PCA reduces the size of the data sample, simplifies the model system, better describes the complex nonlinear system of relationships, and provides a dynamic assessment of the system’s source variables [48]. As a result of this method, new variables (principal components, PCs) were obtained, each of which was a linear combination of the original variables. We used algorithms embedded in the Statistica ver. 12.0 software for PCA calculations. It has been reported that a data normalisation procedure can be used for PCA. To do this, the most often used method is dividing each value by the average for the series of observations or by its standard deviation [100]. This procedure reduces the difference in the original data and simplifies the PCA, but it may lead to the loss of certain information. Considering the specifics of the research being carried out and the nature of the initial data, it was important to consider all changes in the characteristics of the microalgae, so the data were not normalised.

The initial data consisted of measurements of the biochemical and antioxidant characteristics of the *Cc. oleofaciens* CAMU MZ–Ch4 strain under different cultivation conditions. These conditions were associated with variations in the aeration regime and nitrogen and phosphorus concentrations in the nutrient medium. The following indicators were determined for this strain: biomass concentration; chlorophyll *a* and *b*, carotenoid, protein, lipid, retinol, and α-tocopherol contents; GPx, CAT, and SOD activity; and TBA-active product content in the initial homogenate and during LPO induction. Also, such indicators were determined for the following cultivation conditions: nitrogen concentration, phosphorus concentration, N/P ratio in the cultivation medium, and aeration mode. 

The number of factors identified based on statistical processing was determined based on the eigenvalues of the correlation matrix and the percentage of explained variance for each factor. Using the Kaiser criterion, only factors with eigenvalues greater than one were selected [100]. When analysing the PCA model, we considered which variables formed each factor, as well as the absolute values of factor loadings for each variable.

## 5. Conclusions

This study demonstrated that the strain *Cc. oleofaciens* CAMU MZ–Ch4 is promising for biotechnological research and has good prospects as a source of lipids, pigments, proteins, and vitamins. Under various culture conditions, it can accumulate up to 381.03 mg g^−1^ lipids and 283.56 mg g^−1^ proteins. The total content of chlorophyll can reach 6.37 mg g^−1^, carotenoids 2.12 mg g^−1^, retinol 0.16 mg g^−1^, α-tocopherol 0.68 mg g^−1^, phenolic compounds 0.32 mg g^−1^, and ascorbic acid 0.19 mg g^−1^ DW.

The strain exhibits high adaptive potential and stress resistance by activating various branches of the antioxidant protection system and performing general metabolic restructuring. Given the biochemical characteristics of the strain’s biomass, it is a promising natural source of biologically active compounds for feed production to promote methane mitigation from ruminants and for the nutraceutical and pharmaceutical industries.

In future studies, other strategies to optimise cultivation conditions and increase the productivity of *Cc. oleofaciens* CAMU MZ–Ch4 will be tested, including changing lighting conditions, enriching the CO_2_/air mixture, and performing two-stage cultivation.

Future studies will also provide new information about the role of other components (high- and low-molecular-weight antioxidants) in the antioxidant defence system and about the increasing biotechnologically valuable metabolite content of *Cc. oleofaciens* CAMU MZ–Ch4 and other green microalgae.

An analysis of scientific sources indicates that at this time, studies on the growth of microalgae, the synthesis of biotechnologically valuable compounds, and the changes in cell antioxidant defence systems under stress are rare. At the same time, using abiotic stress to activate the antioxidant cell system and change the metabolic rate is one of the popular strategies for activating the synthesis of biotechnologically valuable microalgal compounds. The existing limitation of research in this direction does not allow for the effective elucidation of the metabolic capabilities of microalgae in their biotechnological use context. Therefore, one of the essential tasks in future research is establishing fundamental regulating mechanisms for cell functional activity.

## Figures and Tables

**Table 1:** Contents of lipids, pigments, and proteins and changes in the antioxidant status of the strain *Cc. oleofaciens* CAMU MZ–Ch4 in cultures with different aeration regimes and culture durations (M ± SD, *n* = 3).

Indicator	Option A	Option B	Option C
	Early Stationary Growth Phase (18 Days)	Early Stationary Growth Phase (18 Days)	Late Stationary Growth Phase (74 Days)
Biomass concentration, g L^−1^	1.35 ± 0.034	1.93 ± 0.061	2.21 ± 0.085 *^#^
Ch *a*, mg g^−1^ DW	4.85 ± 0.24	1.75 ± 0.09 *	0.36 ± 0.04 *^#^
Ch *b*, mg g^−1^ DW	1.52 ± 0.10	0.48 ± 0.01 *	0.13 ± 0.01 *^#^
Carotenoids, mg g^−1^ DW	2.12 ± 0.06	1.30 ± 0.08 *	0.64 ± 0.09 *^#^
Ch *a*/Car	1:0.44	1:0.74	1:1.78
Protein, mg g^−1^ DW	283.56 ± 2.12	131.40 ± 12.26 *	87.80 ± 10.26 *^#^
Lipids, mg g^−1^ DW	137.39 ± 4.51	290.71 ± 1.66 *	381.03 ± 3.96 *^#^
Ascorbic acid, mg g^−1^ DW	0.19 ± 0.57	0.08 ± 0.02 *	0.02 ± 0.01 *
Phenolics, mg routine g^−1^ DW	0.32 ± 0.05	0.23 ± 0.04 *	0.13 ± 0.03 *^#^
Retinol, mg g^−1^ DW	0.08 ± 0.01	0.11 ± 0.02 *	0.16 ± 0.011 *^#^
Tocopherol, mg g^−1^ DW	0.49 ± 0.03	0.43 ± 0.05	0.68 ± 0.06 *^#^
SDH, µmol mg^−1^ min^−1^	0.04 ± 0.01	0.14 ± 0.02 *	0.08 ± 0.02 *^#^
GPx, nMol mg^−1^ min^−1^	0.31 ± 0.01	0.55 ± 0.02 *	0.74 ± 0.08 *
CAT, pmol mg^−1^ min^−1^	25.13 ± 0.37	57.43 ± 0.98 *	88.10 ± 4.69 *^#^
SOD, 10 u.unit mg^−1^ min^−1^	2.59 ± 0.17	3.40 ± 0.53 *	2.87 ± 0.23
TBAaP, nMol g^−1^ DW	94.28 ± 6.68	57.74 ± 10.63 *	33.65 ± 9.38 *^#^
TBAaP_in_, nMol g^−1^ DW	192.8 ± 8.64	132.9 ± 5.54 *	55.46 ± 10.93 *^#^
K_AAC_	0.49 ± 0.01	0.43 ± 0.06	0.61 ± 0.4
pH of medium	10.05 ± 0.04	9.92 ± 0.04 *	9.11 ± 0.06 *
O_2_ disolv. in medium	20.57 ± 2.70	14.48 ± 0.28 *	12.2 ± 0.15 *
CO_2_ in medium	0.072 ± 0.001	0.069 ± 0.001	0.044 ± 0.003

Note: Option A—additional aeration bubbling with a speed of 0.8 L min^−1^; Option B—limited aeration bubbling with a speed of 0.1 L min^−1^; Option C—experiment until the late stationary phase (74 days); * the difference is valid concerning the standard BBM medium (control); ^#^ the difference is valid concerning one experimental group at *p* ≤ 0.05.

**Table 2:** Nitrogen and phosphorus concentrations in the experiments.

Indicator	Experiment Options									
	Control	EO1	EO2	EO3	EO4	EO5	EO6	EO7	EO8	EO9
Total nitrogen, g L^−1^	0.0413	0.0384	0.0258	0.0129	0.0064	0.0026	0.00129	0.00077	0.00038	0.00026
Total phosphorus, g L^−1^	0.0534	0.0072	0.0072	0.0072	0.0005	0.0005	0.0005	0.0002	0.0002	0.0002
N/P ratio	0.77:1	5.33:1	3.58:1	1.79:1	12.8:1	5.2:1	2.58:1	3.85:1	1.9:1	1.3:1

**Table 3:** Content of lipids, pigments, and proteins and changes in the antioxidant status of the strain *Cc. oleofaciens* CAMU MZ–Ch4 with different nitrogen and phosphorus concentrations in the nutrient medium (culture time 14 days; M ± SD, *n* = 3).

Parameters	Control	EO1	EO2	EO3	EO4	EO5	EO6	EO7	EO8	EO9
**Pigments**										
Ch *a*, mg g^−1^ DW	3.78 ± 0.21	1.88 ± 0.08 *	2.66 ± 0.11 *^#^	0.3 ± 0.01 *^#^	0.65 ± 0.16 *^#^	0.95 ± 0.04 *	2.03 ± 0.21 *^#^	1.54 ± 0.01 *^#^	0.76 ± 0.01 *^#^	0.55 ± 0.09 *
Ch *b*, mg g^−1^ DW	1.34 ± 0.08	0.67 ± 0.01 *	0.95 ± 0.03 *^#^	0.1 ± 0.01 *^#^	0.22 ± 0.05 *^#^	0.33 ± 0.01 *^#^	0.62 ± 0.06 *^#^	0.48 ± 0.01 *^#^	0.25 ± 0.01 *^#^	0.18 ± 0.04 *
Car, mg g^−1^ DW	1.61 ± 0.07	1.06 ± 0.04 *	1.18 ± 0.03 *	0.39 ± 0.01 *^#^	0.55 ± 0.13 *	0.66 ± 0.04 *	1.3 ± 0.11 *^#^	1.03 ± 0.01 *^#^	0.52 ± 0.01 *^#^	0.4 ± 0.07 *
Ch *a*/Car	1:0.43	1:0.57	1:0.44	1:1.29	1:0.85	1:0.69	1:0.64	1:0.67	1:0.68	1:0.74
**First metabolites**										
Prot., mg g^−1^ DW	220.19 ± 12.01 *	184.51 ± 3.69 *	197.81 ± 3.94 *^#^	156.87 ± 4.33 *^#^	181.32 ± 2.34 *^#^	n/a	n/a	n/a	n/a	n/a
Lipids, mg g^−1^ DW	141.4 ± 4.7	109.1 ± 7.2	110.2 ± 9.3	92.3 ± 9.4 *	132.8 ± 18.9 ^#^	n/a	n/a	n/a	n/a	n/a
**Second metabolites**										
Retinol, mg g^−1^ DW	0.11 ± 0.04	0.13 ± 0.04 *	0.08 ± 0.01 *^#^	0.02 ± 0.002 *^#^	0.027 ± 0.08 *	n/a	n/a	n/a	n/a	n/a
Tocopherol, mg g^−1^ DW	0.25 ± 0.03	0.2 ± 0.07	0.2 ± 0.02	0.13 ± 0.04 *^#^	0.04 ± 0.004 *^#^	n/a	n/a	n/a	n/a	n/a
**Antioxidant system**										
GPx, nMol mg^−1^ min^−1^	2.09 ± 0.15	1.85 ± 0.31	2.46 ± 0.01 ^#^	2.53 ± 0.15	1.73 ± 0.05 ^#^	n/a	n/a	n/a	n/a	n/a
CAT, pmol mg^−1^ min^−1^	26.0 ± 2.0	49.0 ± 2.0 *	48.0 ± 8.1 *	85.0 ± 6.0 *^#^	70.0 ± 3.4 *^#^	94.0 ± 6.0 *^#^	n/a	n/a	n/a	n/a
SOD, 10 u.un mg^−1^ min^−1^	0.24 ± 0.06	0.42 ± 0.15	0.55 ± 0.08	0.93 ± 0.14 *	0.87 ± 0.12 *	1.44 ± 0.31 *^#^	n/a	n/a	n/a	n/a
TBAaP, nMol g^−1^ DW	76.35 ± 6.98	66.36 ± 4.97	63.27 ± 4.93	71.57 ± 4.17	77.4 ± 6.08	n/a	n/a	n/a	n/a	n/a
TBAaP_in_, nMol g^−1^ DW	101.03 ± 7.45	85.46 ± 5.23 *	90.94 ± 4.37	90.94 ± 4.39	102.15 ± 6.48	n/a	n/a	n/a	n/a	n/a
K_AAC_	0.76	0.78	0.7	0.79	0.76	n/a	n/a	n/a	n/a	n/a

Note: The statistical analysis uses a single-factor dispersion analysis of ANOVA. The reliability between the groups is calculated using the Tukey–Kramer posterior test. * The difference is valid concerning the control; ^#^ the difference is valid concerning the previous value at *p* ≤ 0.05. EO1–EO9—experiment options according to Table 2. n/a—not available.

## Data Availability

Data are contained within the article.
